# Transfer RNA-derived fragment production in calves challenged with *Mycoplasma bovis* or co-infected with bovine viral diarrhea virus and *Mycoplasma bovis* in several tissues and blood

**DOI:** 10.3389/fvets.2024.1463431

**Published:** 2024-11-08

**Authors:** Anna K. Goldkamp, Randy G. Atchison, Shollie M. Falkenberg, Rohana P. Dassanayake, John D. Neill, Eduardo Casas

**Affiliations:** Ruminant Diseases and Immunology Research Unit, National Animal Disease Center, Agricultural Research Service, United States Department of Agriculture, Ames, IA, United States

**Keywords:** tRNA, bovine, *Mycoplasma bovis*, bovine viral diarrhea virus, tRNA-derived fragment

## Abstract

Understanding the molecular mechanisms underlying immune response can allow informed decisions in drug or vaccine development, and aid in the identification of biomarkers to predict exposure or evaluate treatment efficacy. The objective of this study was to identify differentially expressed transfer RNA-derived fragments (tRFs) in calves challenged with *Mycoplasma bovis* (*M. bovis*) or co-infected with *M. bovis* and bovine viral diarrhea virus (BVDV). Serum, white blood cells (WBC), liver, mesenteric lymph node (MLN), tracheal-bronchial lymph node (TBLN), spleen, and thymus were collected from Control (*n* = 2), *M. bovis* (MB; *n* = 3), and co-infected (Dual; *n* = 3) animals, and small RNAs extracted for sequencing. An average of 94% of reads were derived from 5` halves and/or 5` tRFs in serum, liver, WBC, TBLN, spleen, MLN, and thymus. The expression of tRFs in lymphatic tissues (MLN, TBLN, Thymus, Spleen) were highly correlated with each other (r ≥ 0.82), but not with serum and WBC. A total of 25 and 65 differentially expressed tRFs were observed in liver and thymus, respectively. There were no differentially expressed tRFs found in other tissues analyzed. Nineteen thymus tRFs were differentially expressed in Dual compared to Control and MB, and the predicted targets of these tRFs were associated with MAPK signaling pathways and ERK1 and ERK2 cascades. The differentially expressed tRFs found in thymus and liver may underlie mechanisms of thymic depletion or liver inflammation previously observed in BVDV. Additional studies should be pursued to investigate differential expression of the predicted tRF targets.

## 1 Introduction

*Mycoplasma bovis* (*M. bovis*) is a highly contagious bacterium that poses significant challenges in cattle health and production. As it is one of the major causes of bovine respiratory disease (BRD), *M. bovis* infection can result in economic losses for producers and can also lead to the development of mastitis, arthritis, and otitis media (1–4). Previous studies have suggested synergistic mechanisms between *M. bovis* and other pathogens, in which co-infection results in severe lung lesions and exacerbates disease in cattle (5–7). Microorganisms often detected in association with BRD include *Pasteurella multocida*, *Mannheimia haemolytica*, bovine respiratory syncytial virus (BRSV), and bovine viral diarrhea virus (BVDV) (1, 6, 7). However, *M. bovis* and BVDV are reported to be some of the most common pathogens in the tissues of animals with chronic, antibiotic-resistant BRD (5, 8). Due to concern of antibiotic overuse and an incomplete vaccination program, research investigating *M. bovis* and co-infection is necessary to better our understanding of the factors that mediate host defense mechanisms.

Extensive research has shown that changes in gene expression and subsequently protein levels underlie cellular responses to bacterial infection and the response of the host can determine the progression of infection (9–13). With advancements in next generation sequencing methods, small non-coding RNAs have emerged as powerful post-transcriptional regulators of gene expression and could also act as novel biomarkers of disease (14). A relatively new class of small non-coding RNA, called tRNA-derived fragments (tRFs), is produced through the cleavage of mature transfer RNAs (tRNAs). These tRFs have garnered attention for their ability to regulate gene expression through displacement of translation initiation factors, interactions with 80S ribosomes, or even in a microRNA (miRNA)-like manner, where they associate with an RNA-induced silencing complex and target the 3` untranslated region (UTR) of messenger RNAs (mRNAs) (15–18). Previous work has shown that tRFs can arise from nuclear and mitochondrial tRNAs (19). These fragments range from 16 to 36 nucleotides (nt) and can be divided into various subtypes depending on the location in which the mature tRNA is cleaved: 5` halves, 5` tRFs, internal-tRFs (i-tRFs), 3` tRFs, and 3` halves (19–21). 5` halves and 3` halves are generally 31–36 nt long and produced from cleavage near the tRNA anticodon, whereas 5` tRFs, 3` tRFs, and i-tRFs are produced through cleavage of D- and T-loops of the tRNA and are 16–30 nt long (21).

Previous work has shown that host small non-coding RNAs can have a role in suppressing viral replication. For example, bta-miR-2904, bta-miR-2411, and bta-miR-29b were reported to inhibit BVDV replication through targeting genes related to protein synthesis, autophagy and apoptosis, respectively (22–24). Although the interactions of host miRNAs during infection have been well defined, the impact of BVDV and *M. bovis* on tRF expression has been poorly characterized and there are a limited number of studies investigating tRF expression in cattle viral diseases. For example, previous work has shown dysregulated tRF expression in blood samples from bovine leukemia virus (BLV), BVDV, and *M. bovis* infected cattle (25–28). 5′ halves derived from tRNA^GlyCCC^ and tRNA^GlyGCC^ were downregulated in serum of cattle challenged with BVDV and 5’ tRFs derived from tRNA^SecUGA^ were upregulated in the blood of animals challenged with *M. bovis*. However, these studies only define circulating tRFs in biological fluids (serum and white blood cells). Nevertheless, a role for tRFs as potential immune signaling molecules during BVDV and *M. bovis* infection has been implicated. A comprehensive investigation of the tRFome across immune related tissues and in co-infected cattle has yet to be conducted.

In the present study, we aimed to identify variation in tRF expression within three treatment groups of calves: Control (*n* = 2), *M. bovis* (MB; *n* = 3), and Co-infected (Dual; *n* = 3). The objective of this study was to define alterations in tRF abundance due to exposure to MB or due to co-infection with MB and BVDV. Therefore, small RNA sequencing was done in thymus, spleen, tracheal-bronchial (TBLN) lymph node, mesenteric lymph node (MLN), liver, serum, and white blood cells (WBC) across all treatment groups.

## 2 Materials and methods

### 2.1 Animal welfare

Animals housed and samples collected for this study were handled in accordance with the Animal Welfare Act Amendments (7 U.S. Code e §2,131 to §2,156). All procedures were approved by the Institutional Animal Care and Use Committee of the National Animal Disease Center (ARS-2016-581). Intravenous injection of sodium pentobarbital was used to euthanize animals following per label dose and the discretion of the clinical veterinarian.

### 2.2 Animal challenge study

The challenge study was done as previously described (29). Eleven Holstein male colostrum-deprived calves that were all 2 months of age were assigned to one of the treatment groups: Control (*n* = 2), bovine viral diarrhea virus (BVDV; *n* = 3), *M. bovis* (MB; *n* = 3), and dual infection with MB and BVDV (Dual; *n* = 3). On day 0, MB calves were challenged with *M. bovis*, BVDV calves with BVDV, and dual infection calves with BVDV. Control calves were given 5 ml of cell culture supernatant of uninfected cells. On day 6, Dual calves were challenged with *M. bovis*. Five milliliters BVDV or *M. bovis* inoculum was intranasally administered to calves using mucosal atomization device (MAD Nasal, Teleflex, Morisville, NC) attached to a 5 ml syringe. For the *M. bovis* inoculum, each calf received a total of 1 × 10^11^ cfu in 5 ml. For the BVDV inoculum, each calf received a total of 5 × 10^6^ TCID50 in 5 ml. At the end of the study, it was found that all calves (besides the BVDV group) were naturally infected with *M. bovis* before the beginning of the experiment, which was based on antibody measurement using ELISA. Therefore, it was decided to remove the BVDV group from future analysis to avoid confounding effects on gene expression.

Seventeen days after the challenge, calves were humanely euthanized by intravenous administration of sodium pentobarbital. Serum, white blood cells (WBC), liver, mesenteric lymph node (MLN), tracheal-bronchial lymph node (TBLN), spleen, and thymus were collected at necropsy, as previously described (29). Serum samples were collected from all calves via jugular venipuncture in SST vacutainer tubes (BD, Franklin Lakes, NJ). White blood cells were collected by venipuncture in PAXgene tubes (PreAnalytiX GmbH, Hombrechtichon, Zurich, Switzerland). All samples were stored in RNAlater (Millipore Sigma, Darmstadt, Germany) and kept at −80°C until RNA extraction.

### 2.3 RNA isolation, library preparation, and sequencing

Total RNA was extracted from all samples with the MagMax™ mirVana total RNA isolation kit (Life Technologies, Carlsbad, CA, United States), as previously described (29). RNA samples were run on an Agilent 2,100 Bioanalyzer Small RNA chip (Agilent Technologies, Santa Clara, CA, United States) to evaluate concentrations of small RNAs ranging from 10 to 40 nucleotides in size.

For each sample, 6 uL of RNA were used as input for the NEBNext Small RNA Library Prep kit (New England Biolabs, Ipswich, MA, United States) as previously described (29). Resulting libraries were purified using the QiaQuick PCR purification kit (QIAGEN, Germantown, MD, United States). Library concentration at 135–170 base pairs was determined using the Agilent 2,100 Bioanalyzer High Sensitivity DNA chip (Agilent Technologies, Santa Clara, CA, United States). Libraries were pooled in equal concentration and size-selected using AMPure XP beads (Bechman Coulter, Indianapolis, IN, United States), which was followed by purification with the QiaQuick PCR purification kit to concentrate the pooled libraries. The pooled library was stored at −20°C until it was sequenced on the Illumina HiSeq 3000 System (1 × 50 bp; Illumina, San Diego, CA, United States).

### 2.4 Small RNA data processing

FastQC (v 0.12.1) was used to assess quality of the sequenced reads (30). Adapter sequence (AGATCGGAAGAGCACACGTCT) was removed, low-quality bases (Phred score < 20) were trimmed, and trimmed reads were discarded based on length (–minimum-length 13 –maximum-length 40) using Cutadapt (v4.0). MINTmap (v1.0) was used for tRF prediction (21). Cytoplasmic tRNA^1^ and mitochondrial tRNA sequences^2^ from the bovine genome (ARS UCD1.2) were retrieved. Mature tRNA sequences were created by removing introns, incorporating discriminator bases at the −1 position, and adding CCA tails with custom scripts. A list of candidate tRF sequences was created by breaking each tRF into all possible substrings that ranged from 16 to 50 nt. A masked genome file was made using bedtools (v 2.30.0) to determine tRNA exclusivity, where tRNA exons were marked with a ‘1’, post-transcriptional tRNA modifications with a ‘2’, and all other regions with a ‘0’ (31). Only tRFs that exclusively mapped to genomic tRNA regions were kept for analysis.

### 2.5 Small RNA data analysis

To evaluate the proportion of several small non-coding RNAs by tissue, bovine miRNA sequences were retrieved from miRbase and bovine piRNA, snRNA, and snoRNA sequences from RNACentral (32, 33). Sequenced reads were aligned to small non-coding RNAs with bowtie2 (v 2.5.2) to compare expression. Sample and tissue level correlations were calculated using the cor function of the basic stats package in R. Correlation and differential expression heatmaps were created using the R package pheatmap. Bar plots of tRF type, parental tRNA, and length distribution were created using the R package ggplot and summary statistics were calculated using the summarySE function of package Rmisc. The R package ggVennDiagram was used to create Venn diagrams. Some tRFs were predicted to be derived from multiple parent tRNAs. To include all possible tRNA sources for the parent tRNA plot, the counts for each tRF were divided by the number of potential parental tRNAs and then CPM-normalized.

Differential expression analysis was conducted using DESeq2 version 1.44.0. Because of the known functions of tRFs derived from the 5` end of tRNAs and their biased expression, we limited our differential expression analysis to only include 5` halves or 5’tRFs. Only tRFs with ≥5 counts per million (CPM) in at least 2 samples per tissue were considered for differential expression analysis. A DESeq dataset object was created using the DESeqDataSetFromMatrix function and the median of ratios method was applied using the estimateSizeFactors function. Subsequently, the normalization factors were applied to our tRF count matrix. Pairwise comparisons were carried out for each treatment (Control vs. MB, MB vs. Dual, and Control vs. Dual) within each tissue and differential expression was based on a negative binomial GLM and Wald test statistics for each gene. The tRFs that had an adjusted *p*-value ≤0.05 were categorized as statistically significant.

For target prediction, the 3’ UTR sequences of all annotated protein-coding genes in the bovine reference genome were retrieved from Ensembl Release 113. Only the tRFs that were significant in Control vs. Dual and MB vs. Dual comparisons in thymus were analyzed for target prediction. Target prediction was done using miRanda 3.3a based on the complementarity of the tRF sequences and the 3’ UTR sequences. Targets with a binding score cutoff ≥160, an energy cutoff ≤ −20 and strict seed sequence complementarity were considered for further analysis. The resulting list of candidate targets was used for functional enrichment analysis with DAVID (34). Gene ontology terms and pathways with a false discovery rate ≤ 0.05 were considered significant.

## 3 Results

### 3.1 Small RNA sequencing

An average of 298 million raw reads were generated for each tissue (Supplementary Table S1). Adapter and quality trimming resulted in an average of 257 million (86.26%) clean reads with Phred scores ≥20 and lengths ranging from 13 to 40 nt within each tissue. Sequences were mapped to several small RNA classes, where an average of 91.87% mapped to miRNAs, tRFs, piwi-interacting RNAs (piRNAs), small nuclear RNAs (snRNAs), or small nucleolar RNAs (snoRNAs) per tissue (Table 1). 58.8, 54.9, 48, and 63.2% of mapped sequences were derived from miRNAs in MLN, TBLN, thymus, and spleen. 47.8, 44.7, 82.9% of mapped sequences were derived from piRNAs in WBC, liver, and serum. The mapped sequences for all tissues, excluding serum, were predominantly derived from piRNA and miRNA. On average, 96.8% of the sequences mapping to tRFs were derived from nuclear tRNAs and 3.2% were derived from mitochondrial tRNAs in liver, MLN, serum, spleen, TBLN, and thymus. WBC displayed higher expression of tRFs derived from mitochondrial tRNAs (27%) compared to other tissues.

**Table 1:** Percentage of trimmed reads mapping to tRNA-derived fragments (tRFs), piwi-interacting RNAs (piRNAs), microRNAs (miRNAs), small nuclear RNAs (snRNAs), and small nucleolar (snoRNAs) in each tissue.

	tRF	piRNA	miRNA	snRNA	snoRNA
WBC	3,073,821 (1.42%)	103,792,873 (47.80%)	102,461,653 (47.19%)	86,794 (0.04%)	7,702,741 (3.55%)
Liver	12,860,198 (8.47%)	67,944,657 (44.74%)	49,114,431 (32.34%)	213,057 (0.14%)	21,735,887 (14.31%)
MLN	1,910,772 (0.75%)	75,425,424 (29.67%)	149,558,739 (58.82%)	347,372 (0.14%)	27,003,709 (10.62%)
TBLN	3,197,656 (1.21%)	83,508,846 (31.66%)	144,804,630 (54.90%)	218,675 (0.08%)	32,046,818 (12.15%)
Thymus	3,693,605 (1.50%)	86,206,293 (34.92%)	118,542,874 (48.02%)	247,639 (0.10%)	38,187,326 (15.47%)
Serum	42,716,320 (15.73%)	224,996,495 (82.86%)	3,264,096 (1.20%)	398,337 (0.15%)	158,068 (0.06%)
Spleen	2,633,627 (1.05%)	63,777,901 (25.54%)	157,908,311 (63.22%)	150,570 (0.06%)	25,285,860 (10.12%)

The total number of mapped reads are listed in white blood cell (WBC), liver, mesenteric lymph node (MLN), tracheal-bronchial lymph node (TBLN), thymus, serum, and spleen. The percentage of reads mapping to each small non-coding RNA are shown in parenthesis. Each row reflects the percentage of small non-coding expressed in the given tissue and sums to 100%.

### 3.2 Profiling of tRNA fragments

**Figure 1:**
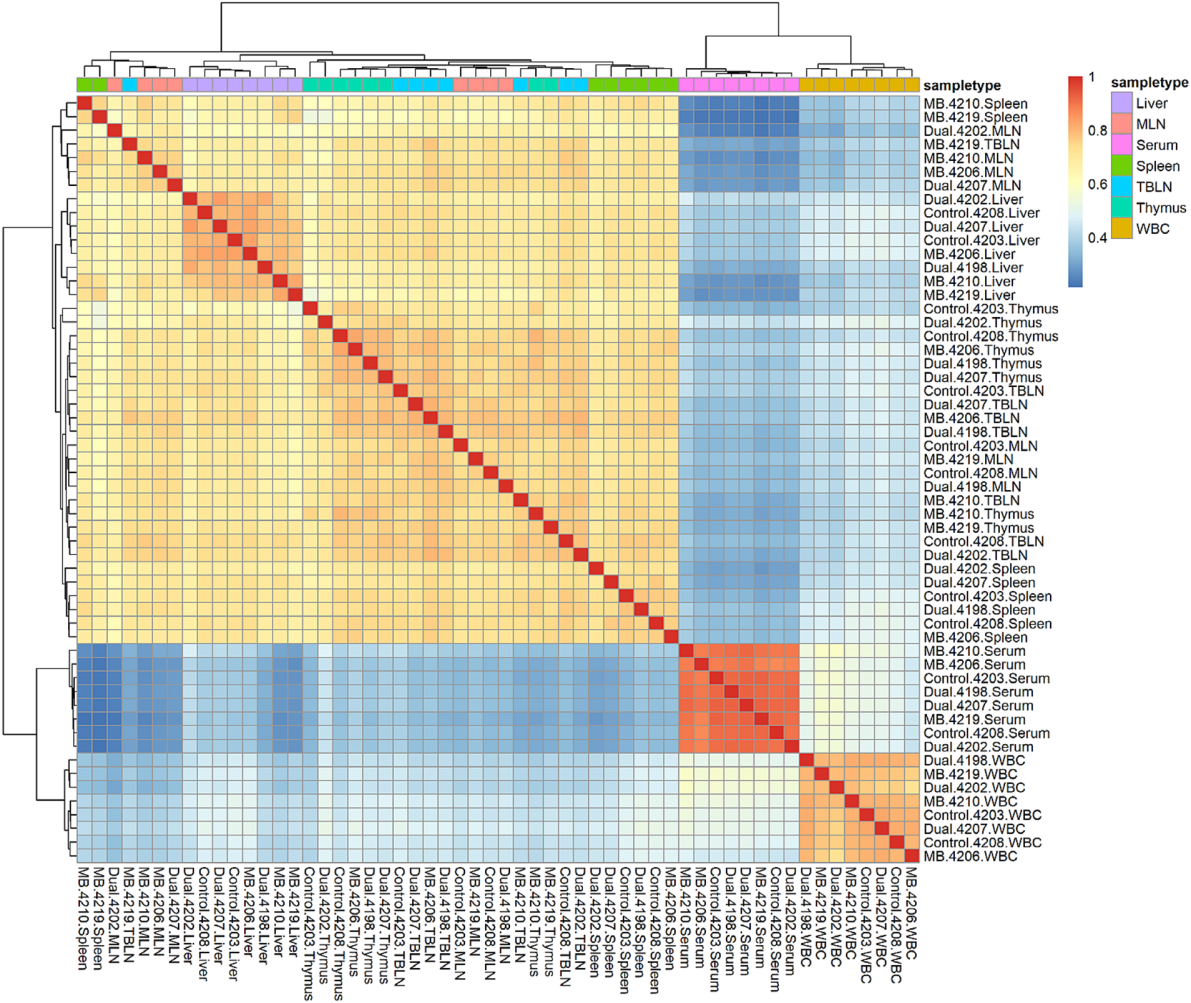
Correlation heatmap plot across all samples and tissues. Red and blue colors represent the highest and lowest correlation values, respectively. Tissues are highlighted in different colors and samples labeled on *x*- and *y*-axis. Pairwise correlation values were calculated using the cor function in base R using the gene expression matrix as input. Samples belonging to liver, mesenteric lymph node (MLN), serum, spleen, tracheal-bronchial lymph node (TBLN), thymus, and white blood cells (WBC) are shown in different colors. Correlations are positive because the majority of expressed tRFs are positively correlated between samples.

A total of 13,485 tRFs were identified (Supplementary Table S2). These tRFs had ≥1 CPM in at least 2 of the 56 samples in the study. The number of tRFs within each tissue is as follows: 9,503 (Liver), 9,063 (Spleen), 8,996 (TBLN), 8,963 (MLN), 8,493 (Thymus), 5,542 (WBC) and 2,421 (Serum). The highest correlations within a tissue were found in serum, WBC, and liver (Figure 1). Spleen, MLN, TBLN, thymus, and liver formed a cluster separate from WBC and serum. Correlations of tRF expression between tissues are shown in Table 2. The expression of tRFs in lymphatic tissues (MLN, TBLN, Thymus, and Spleen) were highly correlated with each other (*r* ≥ 0.82). Liver also shared a high correlation with MLN (*r* = 0.96). However, the correlations between samples derived from biological fluids (WBC and serum) with all other tissues was moderate (*r* = 0.47–0.67).

**Table 2:** Correlation of tRF expression.

	WBC	Liver	MLN	Spleen	TBLN	Thymus	Serum
WBC	1.00	0.48	0.48	0.50	0.47	0.49	0.56
Liver		1.00	0.96	0.82	0.80	0.77	0.57
MLN			1.00	0.86	0.86	0.82	0.59
Spleen				1.00	0.86	0.83	0.57
TBLN					1.00	0.89	0.62
Thymus						1.00	0.67
Serum							1.00

Correlations were calculated across white blood cells (WBC), liver, mesenteric lymph node (MLN), spleen, tracheal-bronchial lymph node (TBLN), thymus, and serum samples using average tRF expression as input with the cor function of R.

**Figure 2:**
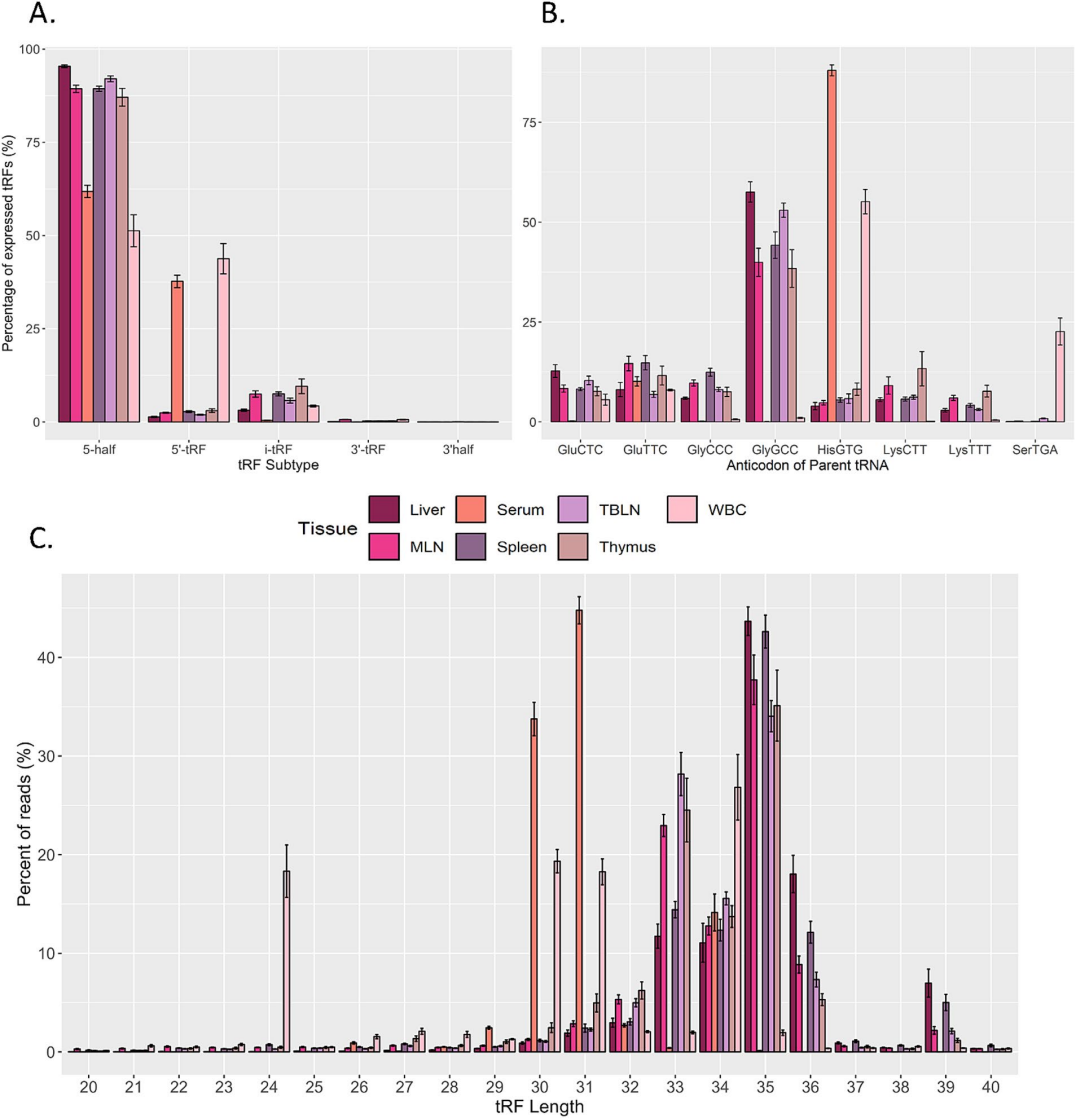
Overview of predicted tRNA-derived fragments (tRF) in the dataset. Distribution of expressed tRFs based on (A) subtype. (B) Parental tRNA source, and (C) length across liver, serum, tracheal-bronchial lymph node (TBLN), white blood cells (WBC), mesenteric lymph node (MLN), spleen, and thymus. CPM-normalized counts were averaged based on size, parent tRNA, or length or each sample in each tissue (n = 8 per tissue). The y-axis represents the proportion of expressed tRFs for each tissue. Summary statistics were calculated using the SummarySE function of the R package Rmisc and standard error bars are shown.

Given that tRFs can have distinct biological roles depending on their origin (5` half, 5` tRF, i-tRF, 3` tRF, and 3` half) and their expression can vary in different physiological states or developmental stages, the expression of tRF subtypes across tissues was characterized (Figure 2A) (15, 35–37). A bias was observed for 5` half and 5` tRF expression, with i-tRF, 3` tRF, and 3` half accounting for less than 10% of tRF expression in all tissues. 5` tRF and 5` half expression was similar in serum and WBC. Conversely, 5` half was the primary subtype expressed in liver, MLN, TBLN, spleen and thymus.

Because tRFs are the processed products of mature tRNAs, the contribution of parental tRNA sources to tRF expression within a sample type was evaluated (Figure 2B). The greatest number of tRFs were produced from tRNA^HisGTG^ in serum and WBC. However, tRNA^GlyGCC^ produced the greatest number of tRFs within tissue samples. Minor contributions (> 5% of total expression in at least one tissue) from other tRNAs were also observed: tRNA^GluCTC^, tRNA^GluTTC^, tRNA^GlyCCC^, tRNA^LysCTT^, tRNA^LysTTT^, and tRNA^SerTGA^. In terms of tRF length distribution, approximately 40% of WBC tRFs were 24 nt or 30 nt in size (corresponding to 5` tRFs) and the remaining fragments in WBC corresponded to 5` halves (31–34 nt; Figure 2C). In serum, the majority of expressed tRFs ranged from 30–31 nt, whereas tRFs in all other tissues were predominantly 33–36 nt in size.

### 3.3 Differentially expressed tRNA-derived fragments

#### 3.3.1 Overview of significant tRFs

A total of 90 tRFs were differentially expressed (adjusted *p*-value <0.05) among treatment groups in at least one tissue (Supplementary Table S3). Of the differentially expressed tRFs, 75 and 15 were 5` half and 5` tRF subtypes, respectively. The parent tRNAs for 47 out of 90 of the significant tRFs were tRNA^GluTTC^, tRNA^GluCTC^, tRNA^LysCTT^, tRNA^GlyTCC^, and tRNA^GlnTTG^. The remaining 43 significant tRFs were derived from 18 different parent tRNAs. The majority of differentially expressed tRFs were upregulated due to co-infection, with 78 out of 90 tRFs upregulated in Dual compared to Control or MB groups.

**Figure 3:**
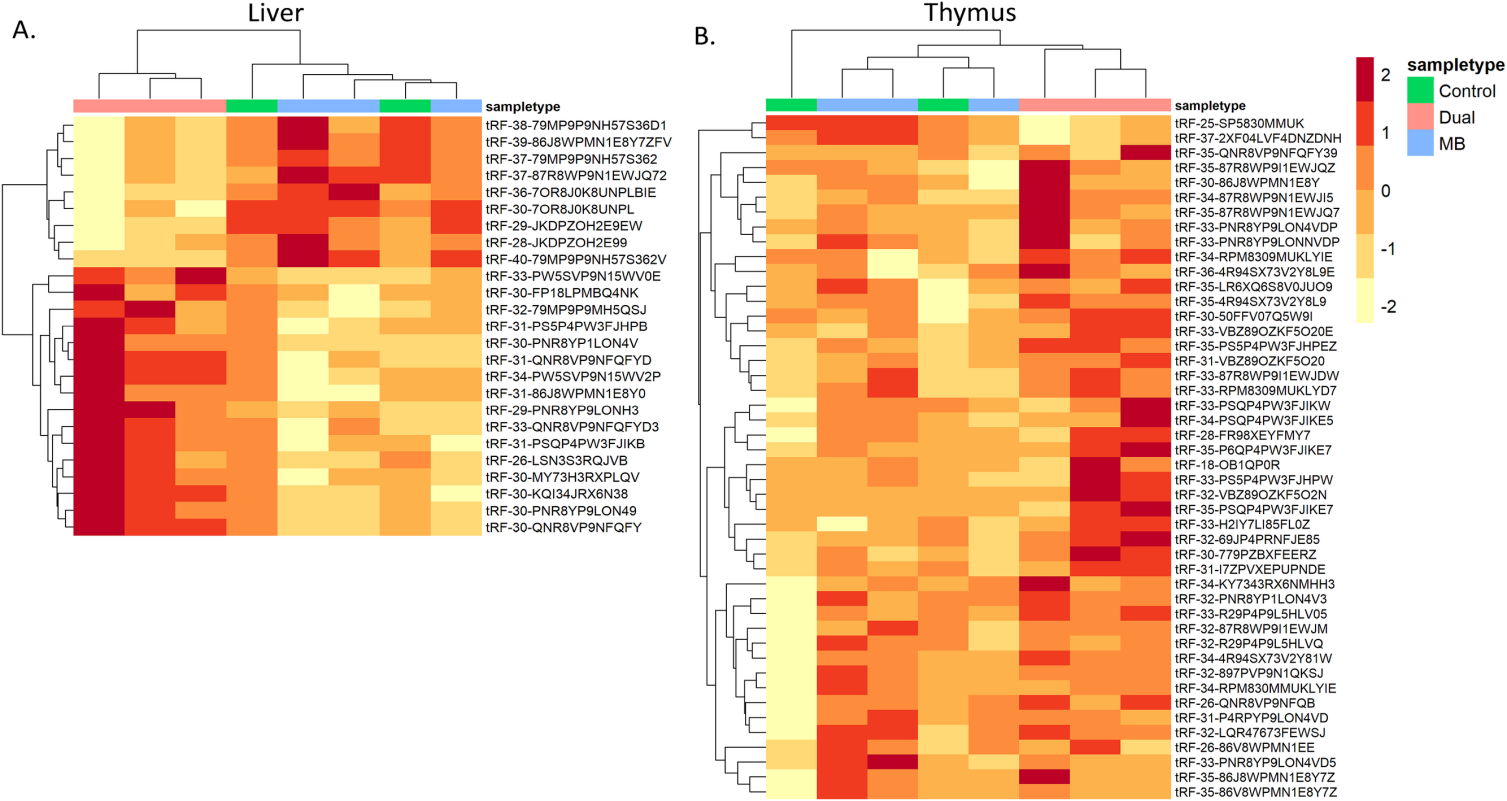
Heatmap of differentially expressed tRNA-derived fragments between Control, *Mycoplasma bovis* (MB), and co-infected (Dual) groups. Log CPM-normalized counts of significant tRFs were plotted in (A) Liver and (B) Thymus. Samples were clustered based on similarity of tRF expression.

Liver and thymus had a total of 25 and 65 significant differentially expressed tRFs, respectively (Tables 3, 4). There were no significant tRFs found in MLN, TBLN, spleen, WBC, or serum. For liver and thymus, clustered heatmaps of differentially expressed tRFs were made to visualize expression across treatments (Figure 3). Samples from the Dual treatment group clustered together based on differentially expressed tRFs in both tissues (Figures 3A,B). This suggests that tRFs in liver and thymus can be used to distinguish infection status. There were no tRFs that were differentially expressed in both liver and thymus. The full DESeq2 output is available in Supplementary Table S3.

**Table 3:** Differentially expressed transfer RNA-derived fragments (tRFs) in Liver.

Comparison	tRF^1^	Subtype	Parent tRNA^2^^,^^3^	log2FC^4^	Padj^5^	Regulation
MB vs. Dual	tRF-30-MY73H3RXPLQV	5’-tRF	GlyTCC	−3.578	5.9E-04	Upregulated in Dual
	tRF-26-LSN3S3RQJVB	5’-tRF	GlyCCC	−3.237	5.9E-04	Upregulated in Dual
	tRF-34-PW5SVP9N15WV2P	5-half	HisGTG	−2.683	8.0E-04	Upregulated in Dual
	tRF-31-QNR8VP9NFQFYD	5-half	GlyTCC	−2.676	8.0E-04	Upregulated in Dual
	tRF-30-QNR8VP9NFQFY	5’-tRF	GlyTCC	−2.383	8.0E-04	Upregulated in Dual
	tRF-30-PNR8YP1LON4V	5’-tRF	GlyCCC	−2.544	3.5E-03	Upregulated in Dual
	tRF-30-PNR8YP9LON49	5’-tRF	GlyCCC	−2.355	5.6E-03	Upregulated in Dual
	tRF-31-PSQP4PW3FJIKB	5-half	LysCTT	−3.101	7.6E-03	Upregulated in Dual
	tRF-31-PS5P4PW3FJHPB	5-half	LysTTT	−2.222	1.2E-02	Upregulated in Dual
	tRF-30-FP18LPMBQ4NK	5’-tRF	iMetCAT	−2.923	1.5E-02	Upregulated in Dual
	tRF-33-PW5SVP9N15WV0E	5-half	HisGTG	−2.027	1.9E-02	Upregulated in Dual
	tRF-40-79MP9P9NH57S362V	5-half	ValCAC	5.295	1.9E-02	Downregulated in Dual
	tRF-37-79MP9P9NH57S362	5-half	ValCAC	1.998	2.5E-02	Downregulated in Dual
	tRF-32-79MP9P9MH5QSJ	5-half	ValAAC	−2.699	2.8E-02	Upregulated in Dual
	tRF-29-JKDPZOH2E9EW	5-half	ProTGG-MT	1.965	2.9E-02	Downregulated in Dual
	tRF-28-JKDPZOH2E99	5’-tRF	ProTGG-MT	2.276	3.5E-02	Downregulated in Dual
	tRF-39-86J8WPMN1E8Y7ZFV	5-half	GluTTC	1.991	3.7E-02	Downregulated in Dual
	tRF-37-87R8WP9N1EWJQ72	5-half	GluCTC	1.764	3.8E-02	Downregulated in Dual
	tRF-33-QNR8VP9NFQFYD3	5-half	GlyTCC	−2.201	3.9E-02	Upregulated in Dual
	tRF-31-86J8WPMN1E8Y0	5-half	GluTTC	−2.080	4.1E-02	Upregulated in Dual
	tRF-30-KQI34JRX6N38	5’-tRF	GluTTC	−4.244	4.2E-02	Upregulated in Dual
	tRF-36-7OR8J0K8UNPLBIE	5-half	LeuTAA-MT	1.730	4.2E-02	Downregulated in Dual
	tRF-29-PNR8YP9LONH3	5’-tRF	GlyCCC	−1.637	4.3E-02	Upregulated in Dual
	tRF-30-7OR8J0K8UNPL	5’-tRF	LeuTAA-MT	1.433	4.9E-02	Downregulated in Dual
	tRF-38-79MP9P9NH57S36D1	5-half	ValCAC	2.497	4.9E-02	Downregulated in Dual

1 tRF = tRNA-derived fragment.

2 tRNA = transfer RNA.

3 MT = Mitochondrial.

4 log2FC = log2 Fold Change.

5 padj = Adjusted *p*-value.

**Table 4:** Differentially expressed transfer RNA-derived fragments (tRFs) in Thymus.

Comparison	tRF^1^	Subtype	Parent tRNA^2^^,^^3^	log2FC^4^	Padj^5^	Regulation
Control vs. Dual	tRF-35-PS5P4PW3FJHPEZ	5-half	LysTTT	−3.61	1.8E-05	Upregulated in Dual
	tRF-32-VBZ89OZKF5O2N	5-half	GlnTTG-MT	−4.87	2.0E-05	Upregulated in Dual
	tRF-33-87R8WP9I1EWJDW	5-half	GluTTC	−3.98	6.2E-05	Upregulated in Dual
	tRF-35-PSQP4PW3FJIKE7	5-half	LysCTT	−4.61	1.2E-04	Upregulated in Dual
	tRF-35-P6QP4PW3FJIKE7	5-half	LysCTT	−5.33	3.2E-04	Upregulated in Dual
	tRF-34-87R8WP9N1EWJI5	5-half	GluCTC	−3.49	3.2E-04	Upregulated in Dual
	tRF-34-4R94SX73V2Y81W	5-half	GluCTC	−5.33	3.3E-04	Upregulated in Dual
	tRF-35-87R8WP9N1EWJQ7	5-half	GluCTC	−3.85	4.0E-04	Upregulated in Dual
	tRF-31-VBZ89OZKF5O20	5-half	GlnTTG-MT	−8.10	6.1E-04	Upregulated in Dual
	tRF-32-LQR47673FEWSJ	5-half	GlyGCC	−4.01	2.6E-03	Upregulated in Dual
	tRF-33-VBZ89OZKF5O20E	5-half	GlnTTG-MT	−4.50	2.8E-03	Upregulated in Dual
	tRF-18-OB1QP0R	5-half	SerGCT-MT	−3.49	2.9E-03	Upregulated in Dual
	tRF-33-PS5P4PW3FJHPW	5-half	LysTTT	−3.10	5.6E-03	Upregulated in Dual
	tRF-33-PNR8YP9LON4VDP	5-half	GlyGCC	−3.43	5.8E-03	Upregulated in Dual
	tRF-35-86J8WPMN1E8Y7Z	5-half	GluTTC	−3.03	6.4E-03	Upregulated in Dual
	tRF-30-50FFV07Q5W9I	5-half	SerTGA-MT	−5.06	9.6E-03	Upregulated in Dual
	tRF-34-RPM830MMUKLYIE	5-half	LeuAAG	−4.81	1.2E-02	Upregulated in Dual
	tRF-34-PSQP4PW3FJIKE5	5-half	LysCTT	−3.18	1.2E-02	Upregulated in Dual
	tRF-32-69JP4PRNFJE85	5-half	ThrTGT	−3.11	1.2E-02	Upregulated in Dual
	tRF-26-QNR8VP9NFQB	5’-tRF	GlyTCC	−3.21	1.2E-02	Upregulated in Dual
	tRF-31-P4RPYP9LON4VD	5-half	GlyGCC	−3.12	1.2E-02	Upregulated in Dual
	tRF-35-4R94SX73V2Y8L9	5-half	GluCTC	−5.17	2.1E-02	Upregulated in Dual
	tRF-36-4R94SX73V2Y8L9E	5-half	GluCTC	−5.25	2.6E-02	Upregulated in Dual
	tRF-35-87R8WP9I1EWJQZ	5-half	GluTTC	−3.17	2.6E-02	Upregulated in Dual
	tRF-33-RPM8309MUKLYD7	5-half	LeuTAG	−6.35	3.1E-02	Upregulated in Dual
	tRF-35-86V8WPMN1E8Y7Z	5-half	GluTTC	−2.36	3.1E-02	Upregulated in Dual
	tRF-28-FR98XEYFMY7	5-half	CysGCA-MT	−5.74	3.3E-02	Upregulated in Dual
	tRF-31-I7ZPVXEPUPNDE	5-half	GlyTCC-MT	−5.12	3.3E-02	Upregulated in Dual
	tRF-32-897PVP9N1QKSJ	5-half	AspGTC	−4.26	3.3E-02	Upregulated in Dual
	tRF-32-R29P4P9L5HLVQ	5-half	AlaCGC	−3.23	3.3E-02	Upregulated in Dual
	tRF-32-87R8WP9I1EWJM	5-half	GluTTC	−2.53	3.3E-02	Upregulated in Dual
	tRF-30-779PZBXFEERZ	5-half	GluTTC-MT	−5.59	4.1E-02	Upregulated in Dual
	tRF-26-86V8WPMN1EE	5’-tRF	GluTTC	−2.19	4.1E-02	Upregulated in Dual
	tRF-25-SP5830MMUK	5’-tRF	LeuCAG	2.98	4.1E-02	Downregulated in Dual
	tRF-33-PSQP4PW3FJIKW	5-half	LysCTT	−2.41	4.2E-02	Upregulated in Dual
	tRF-35-LR6XQ6S8V0JUO9	5-half	LysCTT	−4.54	4.3E-02	Upregulated in Dual
	tRF-34-KY7343RX6NMHH3	5-half	GluCTC	−5.03	4.5E-02	Upregulated in Dual
	tRF-33-PNR8YP9LONNVDP	5-half	GlyGCC	−3.25	4.5E-02	Upregulated in Dual
	tRF-30-86J8WPMN1E8Y	5’-tRF	GluTTC	−2.58	4.5E-02	Upregulated in Dual
	tRF-32-PNR8YP1LON4V3	5-half	GlyCCC	−2.15	4.5E-02	Upregulated in Dual
	tRF-33-PNR8YP9LON4VD5	5-half	GlyCCC	−2.15	4.9E-02	Upregulated in Dual
	tRF-33-R29P4P9L5HLV05	5-half	AlaCGC	−3.29	5.0E-02	Upregulated in Dual
MB vs. Dual	tRF-32-VBZ89OZKF5O2N	5-half	GlnTTG-MT	−4.36	2.55E-05	Upregulated in Dual
	tRF-35-PSQP4PW3FJIKE7	5-half	LysCTT	−4.02	3.45E-04	Upregulated in Dual
	tRF-35-PS5P4PW3FJHPEZ	5-half	LysTTT	−2.64	6.17E-04	Upregulated in Dual
	tRF-35-P6QP4PW3FJIKE7	5-half	LysCTT	−4.25	8.66E-04	Upregulated in Dual
	tRF-32-69JP4PRNFJE85	5-half	ThrTGT	−3.17	2.09E-03	Upregulated in Dual
	tRF-33-H2IY7LI85FL0Z	5-half	GlnTTG-MT	−5.26	2.09E-03	Upregulated in Dual
	tRF-30-50FFV07Q5W9I	5-half	SerTGA-MT	−3.99	2.26E-03	Upregulated in Dual
	tRF-35-87R8WP9N1EWJQ7	5-half	GluCTC	−3.05	3.53E-03	Upregulated in Dual
	tRF-31-I7ZPVXEPUPNDE	5-half	GlyTCC-MT	−5.00	4.39E-03	Upregulated in Dual
	tRF-34-87R8WP9N1EWJI5	5-half	GluCTC	−2.63	4.39E-03	Upregulated in Dual
	tRF-33-PS5P4PW3FJHPW	5-half	LysTTT	−2.75	5.58E-03	Upregulated in Dual
	tRF-30-779PZBXFEERZ	5-half	GluTTC-MT	−5.58	5.58E-03	Upregulated in Dual
	tRF-33-PNR8YP9LON4VDP	5-half	GlyGCC	−2.95	8.58E-03	Upregulated in Dual
	tRF-25-SP5830MMUK	5’-tRF	LeuCAG	3.07	1.47E-02	Downregulated in Dual
	tRF-37-2XF04LVF4DNZDNH	5-half	LysTTT-MT	2.57	2.76E-02	Downregulated in Dual
	tRF-34-RPM8309MUKLYIE	5-half	LeuTAG	−3.18	3.92E-02	Upregulated in Dual
	tRF-34-4R94SX73V2Y81W	5-half	GluCTC	−2.91	3.92E-02	Upregulated in Dual
	tRF-35-QNR8VP9NFQFY39	5-half	GlyTCC	−2.50	3.95E-02	Upregulated in Dual
	tRF-35-87R8WP9I1EWJQZ	5-half	GluTTC	−2.65	4.09E-02	Upregulated in Dual
	tRF-33-R29P4P9L5HLV05	5-half	AlaCGC	−2.99	4.09E-02	Upregulated in Dual
	tRF-34-PSQP4PW3FJIKE5	5-half	LysCTT	−2.46	4.59E-02	Upregulated in Dual
	tRF-33-VBZ89OZKF5O20E	5-half	GlnTTG-MT	−2.75	4.59E-02	Upregulated in Dual
	tRF-31-VBZ89OZKF5O20	5-half	GlnTTG-MT	−3.09	4.84E-02	Upregulated in Dual

1 tRF = tRNA-derived fragment.

2 tRNA = transfer RNA.

3 MT = Mitochondrial.

4 log2FC = log2 Fold Change.

5 padj = Adjusted *p*-value.

#### 3.3.2 Significant tRFs in liver

In liver, MB vs. Dual was the only comparison where significant tRFs were found. 16 of the 25 significant tRFs displayed increased expression in the Dual group compared to MB and all were derived from cytoplasmic tRNAs. Among the nine tRFs that were downregulated in Dual compared to MB, four were derived from mitochondrial tRNAs (Pro^TGG^ and Leu^TAA^; Table 3).

#### 3.3.3 Significant tRFs in thymus

In thymus, 42 and 23 significant tRFs were found between Control vs. Dual and MB vs. Dual, respectively (Table 4). Overall, 41 of 42 tRFs were upregulated in Dual compared to Control and 21 of 23 tRFs were upregulated in Dual compared to MB. Downregulated tRFs included one 5’tRF (*tRF-25-SP5830MMUK*) derived from tRNA^LeuCAG^ with decreased expression in Dual compared to control and MB. Another 5′ half derived from tRNA^LysTTT^, which was downregulated in Dual compared to MB.

**Figure 4:**
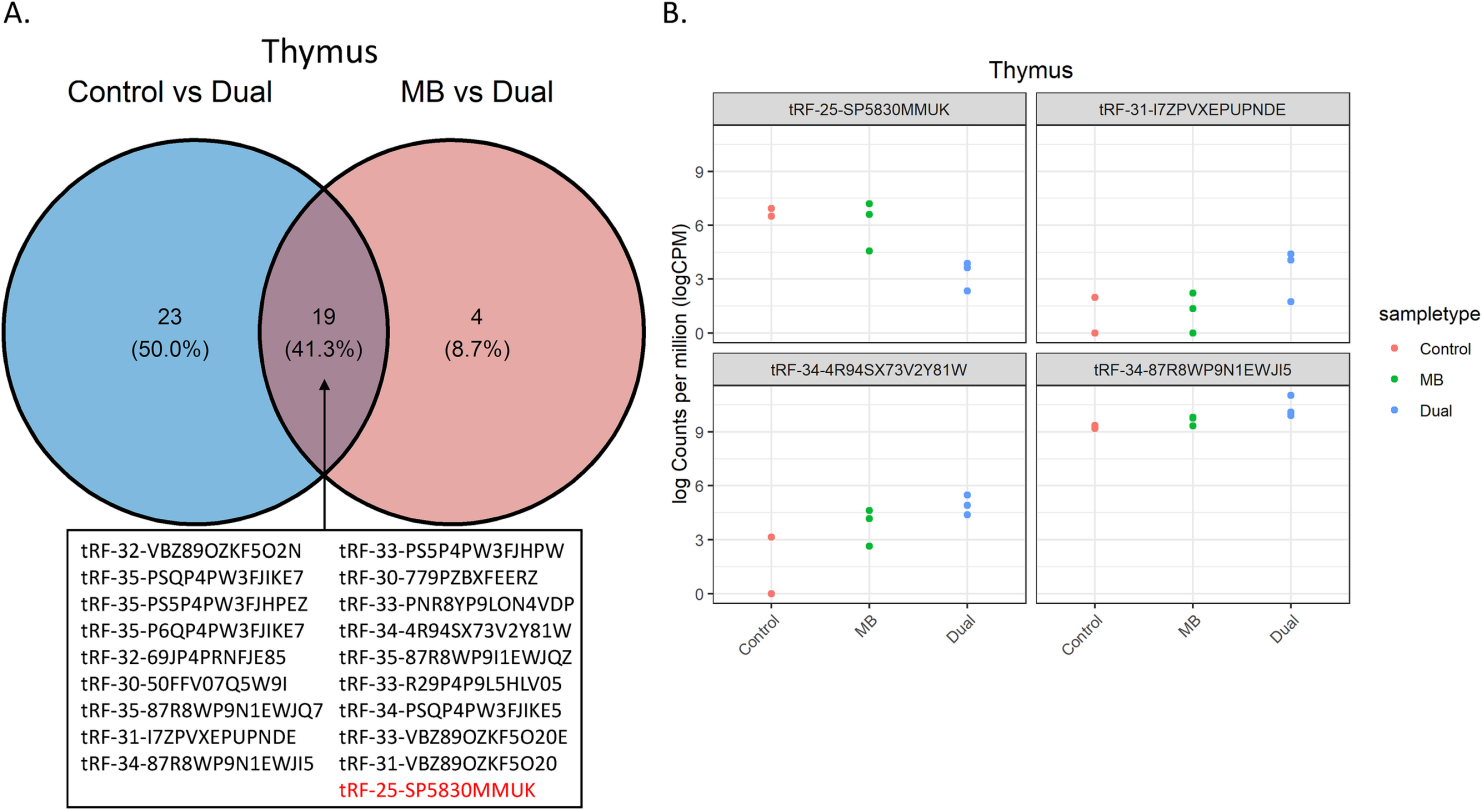
Significant tRFs between Control vs. Dual and MB vs. Dual comparisons in thymus. (A) Venn diagram of the significant tRFs shared between Control vs. Dual and MB vs. Dual comparisons. Upregulated tRFs in Dual compared to Control and MB are shown in black. Downregulated tRF in Dual compared to Control and MB is shown in red. (B) Examples of tRFs with synergistic expression due to co-infection. The y-axis represents log CPM-normalized counts, which were plotted for each replicate across the 3 treatment groups. The x-axis shows the corresponding treatment group. The name of the significant tRF is shown above each plot.

Dual infection may lead to a synergistic effect on gene expression, which may be observed through pairwise comparisons in Control vs. Dual and MB vs. Dual. A venn diagram showed 19 tRFs that were dysregulated in both Control vs. Dual and MB vs. Dual comparisons in thymus. 18 of 19 tRFs were upregulated in the Dual group compared to Control and MB (Figure 4A). The tRFs shared by these two comparisons, such as *tRF-25-SP5830MMUK*, *tRF-31-I7ZPVXEPUPNDE, tRF-34-4R94SX73V2Y81W*, and *tRF-34-87R8WP9N1EWJI5*, had significant alterations in expression specific to the Dual group, suggesting that tRF expression could underlie phenotypes of co-infection (Figure 4B).

#### 3.3.4 Target prediction of significant tRFs in thymus

**Figure 5:**
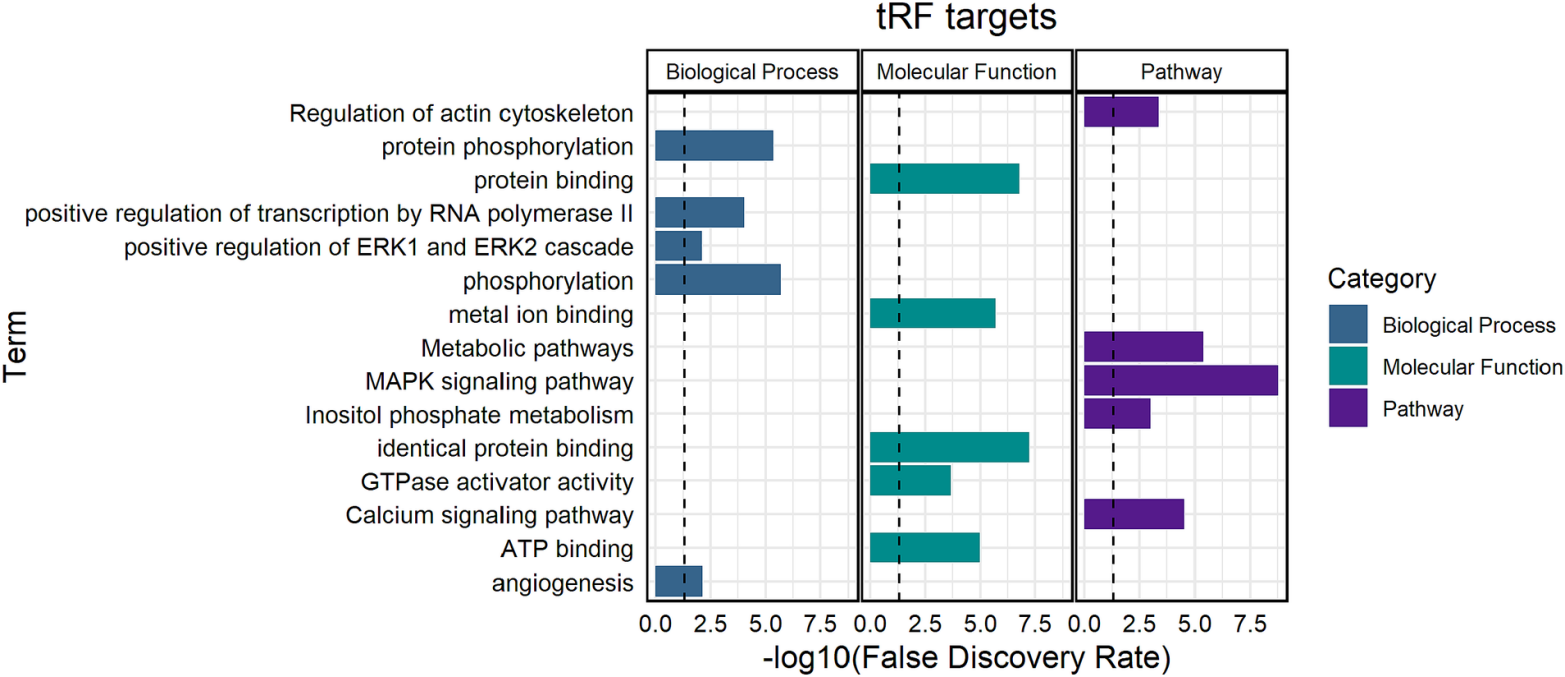
Functional enrichment analysis of significant thymus tRFs. Biological processes, molecular functions, and pathways enriched for tRF gene targets. The black line indicates a false discovery rate (FDR) cutoff of 0.05. The y-axis indicates the term and the x-axis indicates the -log10(FDR).

Target prediction for the 19 synergistic thymus tRFs revealed significant enrichment in mitogen-activated protein kinase (MAPK) signaling pathway, metabolic pathways, and protein binding functions (Figure 5). Enriched biological processes were associated with phosphorylation, positive regulation of transcription, and ERK1 and ERK2 cascades (Supplementary Tables S4, S5).

An upregulated tRF, *tRF-34-4R94SX73V2Y81W*, was predicted to target several MAPK family members (*MAPK4*, *MAPK7*, and *MAPK11*) and MAPK activated protein kinases (*MAPKAPK3* and *MAPKAPK5*). In addition, *tRF-34-4R94SX73V2Y81W*, was predicted to target several elements of the ERK1 and ERK2 cascade, including chemokine ligands 16 and 25 (*CCL16* and *CCL25*) and cluster of differentiation molecules (*CD4*, *CD44*, and *CD74*). Members of the major histocompatibility complex (*BOLA-DOA* and *BOLA-DOB*) were both targeted by *tRF-34-87R8WP9N1EWJI5*, which was upregulated in Dual compared to control and MB. An upregulated 5′ half in Dual, *tRF-31-I7ZPVXEPUPNDE*, was predicted to target the 3′ untranslated region of steroid receptors [nuclear receptor subfamily 2 group C member and nuclear receptor subfamily 3 group C member 1 (*NR3C1*)].

A downregulated tRF in thymus, *tRF-25-SP5830MMUK*, was predicted to target Indian hedgehog (*IHH*), which functions in controlling thymocyte homeostasis, as well as PMS1 homolog 2 (*PMS2*), which is a component of the mismatch repair system.

## 4 Discussion

Diverse tRFs have been identified across cell types and tissues, yet their functional role in gene regulation in different health states continues to be studied (18, 36, 38, 39). It has been established that changes in small non-coding RNA expression can be associated with immunity (28, 40, 41). However, the underlying mechanisms of tRF expression associated with BVDV-host interactions remains poorly understood. The present study provides tRF expression profiles in several tissues during infection with *M. bovis* or co-infection with *M. bovis* and BVDV.

In this study, more than 90% of expressed tRFs were derived from either 5` tRF or 5` half subtypes. These findings are similar to other reports across bovine liver, serum, and white blood cells, where tRFs derived from the 5` end were by far the most abundant subtype (25, 42, 43). Aside from studies in cattle, biased expression of 5` tRNA halves has been observed frequently in human and mouse (44, 45). In mice, 5` halves are highly expressed in hematopoietic and lymphoid tissues compared to other tissues and can also be identified in serum more abundantly than miRNAs (44, 46, 47). When evaluating the abundance of different small non-coding RNA classes in serum in the present study, biased expression of tRFs (15.73%) was found compared to miRNAs (1.20%), snRNAs (0.15%) and snoRNAs (0.06%). In all remaining sample types, tRFs only had greater expression compared to snRNAs. This could suggest a higher involvement of tRFs in various cellular processes. Although piRNAs are generally highly abundant in germ cells, these results suggest that piRNAs are abundant in certain somatic tissues and may have functions outside of the germline. For serum and WBC, tRF expression displayed the lowest correlations with lymphatic tissues, which may indicate that circulating tRFs are not intimately connected with host response. It is possible that certain tRFs are co-regulated across lymphoid tissues and are involved in shared immune responses. Previous work evaluating altered miRNA expression due to *M. bovis* and BVDV also found that lymphatic tissues were uncorrelated with samples from blood (29). In previous work and the current study, WBC and serum had the fewest differentially expressed miRNAs and no differentially expressed tRFs in experimentally infected animals. Together, this seems to indicate that circulating tRFs in WBC and serum are not promising biomarkers in animals exposed to BVDV and *M. bovis*.

Following respiratory syncytial virus infection (RSV) in humans, it was found that the induction of tRF expression was virus specific, where RSV resulted in increased expression of 5` halves derived from tRNA^GlyCCC^ yet human metapneumovirus did not impact expression of this tRF (38). Previous studies have also found that tRFs from tRNA^GlyCCC^ are downregulated in the serum of calves challenged with BVDV and in liver from patients with advanced hepatitis B and C infection (26, 48). In liver and thymus, all significant tRFs derived from tRNA^GlyCCC^ were upregulated in the co-infected group in this study. Although there is variation in the direction of regulation of tRFs derived from tRNA^GlyCCC^, they appear to often be dysregulated due to infection and a role in viral replication has been implicated (26, 48–50). Differentially expressed tRFs derived from tRNA^GlyCCC^ in this study may be related to immune evasion by BVDV to ensure successful viral replication, where upregulated tRFs promote replication by inhibiting host defense genes. In addition, infection induced cleavage of tRNA^GlyCCC^ may also result in modulation at the translational level (42, 51, 52). Additional studies should be done to evaluate the relationship between tRFs and mature tRNA abundance in the context of *M. bovis* and/or BVDV infection.

Similar to miRNAs, differentially expressed tRFs can distinguish co-infected groups in liver and thymus. Differential expression among treatment groups showed that 75 of 90 tRFs were 5` halves and thymus showed the most differential expression between treatments, which further supports a role for 5` halves in immune response. A small portion of tRFs were downregulated due to co-infection in the current study, where 9 of 25 and 2 of 23 tRFs had decreased expression in Dual compared to MB for liver and thymus, respectively. Potentially, a reduction in tRF counts in the co-infected group could be associated with depleted lymphocytes. Furthermore, 18 of 19 tRFs synergistically expressed tRFs were upregulated in Dual compared to Control and MB groups in thymus. Since the control and MB groups displayed similar expression profiles of these tRFs, their disrupted expression in the dual group could underlie an increased pathogen virulence or greater symptom severity due to co-infection.

The tissue with the greatest number of differentially expressed tRFs among treatments was thymus. Thymus is a lymphatic tissue that is often reported as a targeted organ during infection (53). Thymus-associated lymphoid depletion has been observed in cattle infected with BVDV, which can be accompanied by decreased lymphocyte proliferation (54, 55). The molecular mechanisms of thymic depletion are not well understood, but it has been suggested that infection induces an increase in glucocorticoid hormone levels that can cause steroids to trigger apoptosis in thymocytes (53). In mice, it was found that tRNAs are capable of binding to glucocorticoid receptors and other work in rats has shown miRNAs can bind to the 3` UTR of glucocorticoid receptors to control glucocorticoid responsiveness (56, 57). Target prediction revealed that *NR3C1*, which is a glucocorticoid receptor, was targeted by an upregulated tRF in the Dual group, *tRF-31-I7ZPVXEPUPNDE*. It is possible that binding of differentially expressed tRFs in thymus could regulate glucocorticoid receptor activity and influence glucocorticoid signaling, which may contribute to depletion of the thymus.

Target prediction also showed that MAPK pathways and ERK1 and ERK2 cascades may be impacted by significant thymus tRFs in the Dual group compared to Control and MB. The MAPK pathway acts as a signaling cascade that plays a role in T cell differentiation. Several molecules in the MAPK pathway (*MAPK4, MAPK7*, *MAPK11, MAPKAPK3*, and *MAPKAPK5*) were the predicted targets of *tRF-34-4R94SX73V2Y81W* in the Dual group, which could lead to inhibited MAPK signaling and subsequently reduce T cell development. In addition, the MAPK pathway plays a role in apoptosis which could increase cell death and reduce the number of functional T cells (58, 59). *CCL25*, which is a chemokine receptor that functions in guiding thymocyte migration, may also be inhibited by *tRF-34-4R94SX73V2Y81W* (60, 61). Fewer precursor cells migrating to the thymus could reduce T cell output and impact thymic architecture, which may be a potential mechanism involved in thymic atrophy.

In a previous study, it was found that *IHH* can act as a negative regulator of thymocyte development in mice and *IHH* was the predicted target of *tRF-25-SP5830MMUK,* which was downregulated in co-infected animals. Perhaps, infection-associated thymic atrophy leads to downregulation of *tRF-25-SP5830MMUK*, which upregulates *IHH* and causes thymocyte loss (62, 63).

Although studies have demonstrated that lymph node, spleen, and liver are preferred sites of viral replication for BVDV, significant tRFs were found only in the liver (64). Previous work identified an upregulated microRNA, miR-122, which was liver-specific and enhanced the replication of hepatitis C (65). Upregulated tRFs in the liver of the Dual group could behave similarly to miR-122 and increase replication of BVDV.

The liver is not often considered as an important organ in the context of BVDV pathogenesis, yet it plays a pivotal role in inflammatory response. In the present study, two liver tRFs (*tRF-34-PW5SVP9N15WV2P* and *tRF-33-PW5SVP9N15WV0E*) that were upregulated in co-infected animals compared to MB were 5′ halves derived from tRNA^HisGTG^. A previous study found that an abundance of 5′ halves derived from this tRNA could stimulate an immune response to mycobacterial infection and lead to activation of Toll-like receptor 7 (*TLR7*) to induce cytokine production (35). This could suggest that enhanced *TLR7* signaling due to increased expression of *tRF-34-PW5SVP9N15WV2P* and *tRF-33-PW5SVP9N15WV0E* could lead to infection-trigged inflammation in the liver of animals exposed to BVDV and *M. bovis* (66).

We must acknowledge that there are limitations to the current study. The present work included small sample sizes which may not account for variation in immune response. There was also an unintentional natural infection of Control, MB and Dual groups which may have caused the lack of differential expression between Control and MB groups. The BVDV-2A strain was used in this study and it should be noted that use of BVDV1 subtypes may elicit a different immune response and subsequently lead to differences in tRF expression. Differential expression of tRFs can be a useful tool when integrated with mRNA expression profiles to determine candidate gene targets that are dysregulated in infected animals. By characterizing the mRNAs that are regulated by differentially expressed tRFs, the functional roles of tRFs and their potential involvement in immune related pathways can be established. Future work will include validating the dysregulation of the tRF gene targets and determining the mechanistic connection between tRF expression and *M. bovis* and BVDV co-infection.

The expression data demonstrates distinct host response patterns to either *M. bovis* or co-infection. By examining the expression profiles of small non-coding RNAs in immune-related tissues during infection, candidate tRFs have been identified to assess association with pathogen survival. These molecules may selectively influence pathways crucial for mycobacterial pathogenesis and serve as candidates to improve animal health. However, further investigation is required to establish their regulatory targets and assess their potential as diagnostic markers of exposure.

## Data Availability

The original contributions presented in the study are publicly available. This data can be found here: NCBI BioProject, accession PRJNA530924.

## Footnotes

^1^ http://gtrnadb.ucsc.edu

^2^ http://mttrna.bioinf.uni-leipzig.de

## Author’s note

R.A. and S.F. completed all work as National Animal Disease Center, USDA-ARS employees. The work of R.A. predates his employment at the Center for Veterinary Biologics, USDA-APHIS. The work of S.F. predates her employment at Auburn University College of Veterinary Medicine.
